# Clinical features of visual migraine aura: a systematic review

**DOI:** 10.1186/s10194-019-1008-x

**Published:** 2019-05-30

**Authors:** Michele Viana, Erling Andreas Tronvik, Thien Phu Do, Chiara Zecca, Anders Hougaard

**Affiliations:** 1 Headache Center, Neurocenter of Southern Switzerland (NSI), Regional Hospital Lugano, Via Tesserete 46, 6901 Lugano, Switzerland; 2 Headache Group, Department of Basic and Clinical Neurosciences, King’s College London, London, UK; 3 Department of Neuromedicine and Movement Science, Norwegian University of Science and Technology, Trondheim, Norway; 4 Norwegian Advisory Unit on Headache, St. Olavs University Hospital, Trondheim, Norway; 5 Danish Headache Center, Department of Neurology, Rigshospitalet Glostrup, Glostrup, Denmark; 6 Faculty of biomedical Sciences, Università della Svizzera Italiana, Via Buffi 13, 6900 Lugano, Switzerland

**Keywords:** Migraine aura, Migraine with aura, Visual symptoms, Visual disturbances, Scotoma, Clinical features, Scintillating scotoma, Zigzag lines, Blurred vision

## Abstract

**Background:**

Migraine aura (MA) is a common and disabling neurological condition, characterized by transient visual, and less frequently sensory and dysphasic aura disturbances.

MA is associated with an increased risk of cardiovascular disorders and is often clinically difficult to distinguish from other serious neurological disorders such as transient ischemic attacks and epilepsy. Optimal clinical classification of MA symptoms is important for more accurate diagnosis and improved understanding of the pathophysiology of MA through clinical studies.

**Main body:**

A systematic review of previous prospective and retrospective systematic recordings of visual aura symptoms (VASs) was performed to provide an overview of the different types of visual phenomena occurring during MA and their respective frequencies in patients. We found 11 retrospective studies and three prospective studies systematically describing VASs. The number of different types of VASs reported by patients in the studies ranged from two to 23. The most common were flashes of bright light, “foggy” vision, zigzag lines, scotoma, small bright dots and ‘like looking through heat waves or water’.

**Conclusions:**

We created a comprehensive list of VAS types reported by migraine patients based on all currently available data from clinical studies, which can be used for testing and validation in future studies. We propose that, based on this work, an official list of VAS types should be developed, preferably within the context of the International Classification of Headache Disorders of the International Headache Society.

## Introduction

Migraine with typical aura is a highly prevalent disorder as it affects 8% of the general population [1]. Typical migraine aura (MA) symptoms are completely reversible visual, sensory, or language disturbances. Visual aura symptoms (VASs) are by far the most common and occur in 98–99% of MAs, whereas disturbances of sensation and language occur in 36% and 10% of auras, respectively [2]. In addition to being the most common aura symptoms, VASs are also the most multifaceted. In clinical studies of VAS, patients have reported a plethora of different, often complex, visual disturbances. Viana and colleagues previously observed that these visual phenomena could be effectively defined by subdividing the perceived visual scenarios into so-called elementary visual symptoms (EVS), such as zigzag lines, crescent shapes, and flickering lights [2].

Several studies have investigated the clinical features of VASs but so far there is no consensus regarding which different types of EVSs occur during MA and there is no agreement on the terminology that should be used to describe EVSs.

While MA is likely caused by cortical spreading depression, a transient wave of neuronal depolarization of the cortex [3–5], there is currently no pathophysiological explanation for the marked heterogeneity of visual symptoms. An accurate description of the clinical features, in combination with investigations such as neuroimaging, is necessary to provide a better understanding of the underlying mechanisms.

Even more importantly, there are serious clinical issues related to MA that call for improved characterisation of the individual features. MA is associated with an increased risk of ischemic stroke [6, 7], atrial fibrillation [8], and patent foramen ovale [9]. It is also often clinically challenging to differentiate MA from other conditions, particularly transient ischemic attacks [10] and occipital epilepsy [11, 12]. Improved clinical characterisation of MA will likely improve the diagnostic accuracy and identification of patient subgroups at risk of comorbidity.

The aim of this article was to review all published studies providing systematic descriptions of VASs, with information on how frequently different EVSs are reported by MA patients. Based on these data, we aimed to create a comprehensive list of the different types of EVSs including their respective frequency of occurrence.

## Methods

### Literature search

The literature search was performed on June 1st 2018. We used the PubMed/MEDLINE database to identify published studies systematically investigating the clinical features of visual aura. The search string was (((“migraine with aura”[Title/Abstract]) OR “migraine aura”[Title/Abstract]) AND visual [Title/Abstract]). The bibliographies of all included studies were also searched as well as literature that was known to be relevant by the authors. Moreover, we considered the bibliography of the International Classification of Headache Disorders, Third Edition (ICHD-3) [13].

Inclusion criteria were a minimum of 10 migraine patients included where the features of VASs were described. We put this cut-off as generally small case series typically focus on unusual case presentations and are not able to provide an externally valid spectrum of manifestations. We felt that 10 was a reasonable number, although this was an (expert) agreement and not based on scientific evidence. In addition, only articles in English were considered. We excluded studies focusing exclusively on the description of visual disturbances relative to high-tier areas (i.e. prosopagnosia or dyschromatopsia). Furthermore, we did not consider articles exclusively relating to familial or sporadic hemiplegic migraine, basilar-type migraine, persistent auras or symptomatic (secondary) MA.

### Data extraction

Two authors independently reviewed the abstracts found in the literature search (MV, AH). If the title or abstract indicated relevant data, the entire manuscript was examined. Any disagreement between the two authors was resolved by consensus by involving a third person (ET). From included articles, we extracted for the following data categories: publication information (authors, years), population (number of patients), number of auras recorded, study methodology, description of visual aura disturbances, and their frequencies of occurrence on the total number of auras recorded.

### Procedure of composition of the list of EVSs

We created a list of all EVSs with their respective frequency of occurrence (minimum and maximum values found in the studies). In case of discrepancy in the terminology for a given EVS, we reported all definitions. In the list, we included only EVS, and not any visual symptoms described by a combination of two or more EVSs (e.g. “scintillating scotoma”).

## Results

**Fig. 1:**
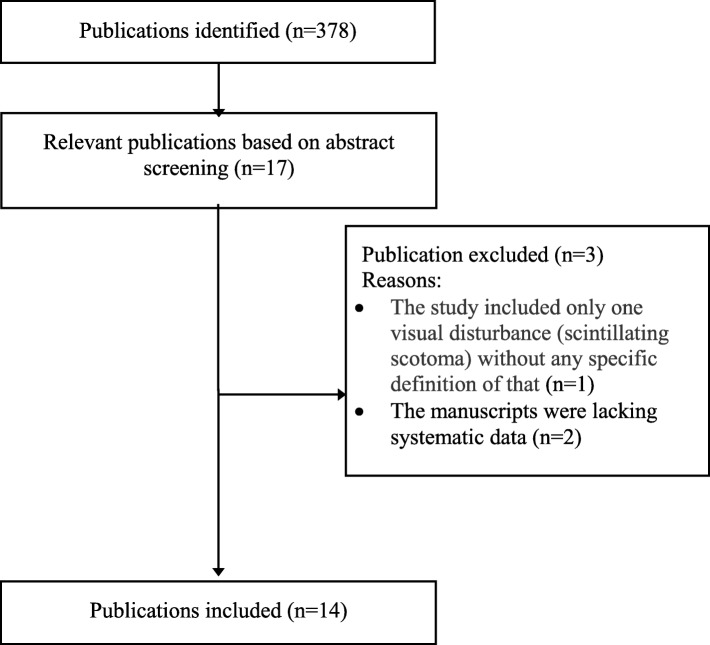
Flowchart of the review process

The search strategy identified 378 published studies (Fig. 1). Seventeen papers fulfilled our case definition [2, 14–29]. One study was excluded since “scintillating scotoma” (79.3%) was the only VS reported [27]. It is likely that such description (not specified in the paper) was used to indicate any visual aura symptom, as no other type of visual disturbances were reported. We also excluded two manuscripts due to data not being systematically presented and since it was impossible to extract the frequency of occurrence of visual disturbances [28, 29]. Key findings of the remaining 14 studies are summarized in Table 1.

**Table 1:** Studies found in literature describing systematically aura disturbances in a population

STUDY	Patients #	Auras #	Prospective	Visual Disturbances #	Quality of Elementary Visual Disturbances (prevalence on total auras)
Viana 2017 [2]	72	216	Y	20	Flashes of bright light (30%), ‘foggy’ / blurred vision (25%), zigzag or jagged lines (24%), scotoma (23%), phosphenes (small bright dots) (19%), flickering light (12%), ‘like looking through heat waves or water’ (8%), visual snow (7%), white spots (7%), ‘bean-like’ forms like a crescent or C-shaped (7%), hemianopsia (6%), deformed images (alteration of line / angles) (6%), ‘tunnel’ vision (4%), curved or circular lines (4%), colored dots / spots of light (3%), black spots (3%) oscillopsia (2%), fractured vision (1%), anopia (1%), alteration of the perception of distance (1%).
Hansen 2016 [14]	267	251	N	4	Dots or flashing lights (70%), wavy or jagged lines (47%), blind spots (42%), tunnel vision (27%).
Petrusic 2014 [15]^a^	40	40	N	4	Scintillating scotoma (67%); zigzag lines (25%); blurred vision (60%); tunnel vision (40%).
Queiroz 2011 [16]	122	122	N	23	Blurred vision (54%), small bright dots (47%), zigzag or jagged lines (41%), flashes of bright light (38%), blind spots (33%), flickering light (30%), ‘like looking through heat waves or water’ (24%), hemianopsia (24%), white spots (22%), coloured dots/spots of light (19%), corona phenomena (18%), curved or circular lines (18%), small black dots (17%), ‘bean-like’ forms like a crescent or c-shaped (16%), black spots (14%), like a mosaic (13%), things look farther away than they really are (13%), round forms (12%), ‘tunnel’ vision (9%), micropsia (4%), things look closer than they really are (3%), macropsia (things look larger than they really are) (3%), complex hallucinations (3%)
Sjaastad 2006 [17]	178	233	N	12	Scintillating scotoma (a propagating “crescent” of the homonymous type) of the homonymous type (62%), obscuration (“dimness”/ foggy vision) (33%), photopsia (“unformed flashes of light”/star-shaped figures) autokinesis (4%), depth vision failure (3%), autokinesis (4%), depth vision failure (2%), tunnel vision (2%), anopia (2%), metamorphopsia (2%), hemianopsia (2%), micropsia (2%), macropsia (1%).
Eriksen 2004 [18]	362	362	N	4	Flickering light (91%), zigzag lines (fortification) (57%), scotoma (70%), preserved central vision (12%).
Cologno 2000 [19]	64	340	Y^a^	3	Scintillating scotomas and fortification spectra (38%), scintillating scotomas (29%) or fortificating spectra (27%).
Kallela 1999 [20]	321	NR	N	4	Hemianopsia (31%), scintillating scotoma (57%), photopsia (52), blurring vision (34%),
Mattson 1999 [21]	68	NR	N	2	Zigzag line (29–35%), scintillation (54–59%)
Queiroz 1997 [22]	100	100	N	18	Small bright dots (“stars”) (42%), White spots/flashes of light (photopsias) (39%), teichopsia (20%), other zigzag lines (17%), coloured spots of light (15%), other lines (curves, straight, etc) (9%), “Blind spots” (scotomata) (32%), black dots/spots (13%), hemianopsia (6%), “foggy”/blurred vision (27%), “as looking through heat waves/water” (10%), “tunnel vision” (10%), “mosaic”/fractured vision (6%), micropsia/macropsia/teleopsia (2%), corona phenomena (2%), complex hallucination (1%), “slanted vision” (1%), “like a negative of film” (1%).
Russel 1996 [23]	161	163	N	4	Flickering light (87%), zigzag lines (fortification) (81%), scotoma (50%), preserved central vision (22%),
Lanzi 1994 [24]	47	47	N	4	Foggy vision (53%), scintillating scotomas (41%), negative scotomas (5%), white spots (5%)
Russel 1994 [25]	20	56	Y	3	A scotoma occurred in 15 attacks (26%), Visual disturbances flickering in 39 attacks (69%). 26 attacks of a semicircular zigzag line (fortification). 1 attack had small flickering star-shaped figures. In 5 attacks the light was constant with zigzag lines. Visual snow, as well as several small scotomas merging to one scotoma was also reported.
Hachinski 1973 [26]^b^	100	100	N	6	Binocular visual impairment (transient blindness and blurring of vision the most common ones) and/or scotomas (77%), distortion and hallucination (micropsia and macropsia were the most common, inversion, alteration of the perception of motion and elaborate hallucination were less seen) (16%), uniocular visual impairment and scotoma (7%)

^a^ This study was conducted in a population of teenagers. ^b^ This study was conducted in a population of children

In Table 2, we report a list of EVSs that have been described in at least one study. The minimum and maximum frequency of occurrence of each EVS is also provided here. “Scintillating scotoma” was not reported in the list as this it is a combination of two or more EVSs, i.e. “scotoma” and “zigzag or jagged lines” and/or “flickering light”.

**Table 2:** List of Elementary Visual Symtpoms (EVSs)  of migraine aura as reported in literature and the range of their frequency in the studies

Elementary Visual Symptoms of aura	Frequency (range %)
1. Flashes of bright light / unformed flashes of light / star-shaped figures	16–38
2. ‘Foggy’/blurred vision or “dimness”	25–54
3. Zigzag or jagged lines	24–81
4. Scotoma	23–77
5. Blind spots (scotomata)	32
6. Black dots	3–17
7. Phosphenes (small bright dots)	19–70
8. Flickering light	12–91
9. ‘Like looking through heat waves or water’	8–24
10. Visual snow	7
11. White Spots	7–22
12. ‘Bean-like’ forms like a crescent or C-shaped	7
13. Hemianopsia	6–24
14. Deformed images (alteration of line/ angles) / Metamorphopsia	2–6
15. ‘Tunnel’ vision	4–27
16. Curved or circular lines	4–18
17. Round forms	12
18. Colored dots / spots of light	3–19
19. Oscillopsia /autokinesis (movement of stationary objects)	2–4
20. Like a mosaic	13
21. Fractured Vision	1
22. Corona phenomena	2–18
23. Anopia	1–2
24. Things look farther away than they really are	1–13
25. Things look closer than they really are	1–3
26. Macropsia (things look larger than they really are)	1–3
27. Micropsia (things look smaller than they really are)	2–4
28. “Like a negative of film”	1
29. “Slanted vision”	1
30. Complex hallucinations	1–3

The total number of EVSs was 30. The frequency of each EVS varied from 1% to 91%. Some EVSs were reported in one paper only (i.e. complex hallucinations, “slanted vision”, “like a negative film” [16]) while others were reported in the majority of studies (flickering lights, bright light, zigzag lines, scotoma/hemianopsia).

## Discussion

We systematically reviewed studies of VASs in order to create a list of all visual features reported during MA. We identified 14 studies, of which only three were prospective. The low number of prospective studies is a major limitation as the complex and polyhedral manifestations of MA are difficult to recall retrospectively.

### Main findings

First, there is a high variability in the number of VASs used in each study, varying from two to 23. The majority of the studies subdivide the VASs into four types (but not the same combination of four). Only in four studies, the disturbances were separated into more than 10 entities.

Second, in some papers, some of the EVSs are merged into one unique entity (e.g. “scintillating scotoma”), although these represent combinations of features that can be individually experienced during an aura (e.g. “scotoma” and “zigzag or jagged lines”/ “flickering lights”) [2].

Third, in some cases the description of a particular VAS is not in line with the rest of literature (e.g. in one study “scintillating scotoma” is described as “a propagating “crescent” of the homonymous type” [17] without any negative visual symptom).

Fourth, some descriptions of EVSs are not sufficiently unequivocal and specific. For example, what is the exact difference between “blind spots” and “black dots”? Does this depend on the size of the area(s) of the visual field involved, or the quality of the EVS (i.e. a blind area versus a black area)?

Fifth, some descriptions are related mostly to a feature of EVSs more than a given EVS. Indeed, this is the case of “flickering lights” which can be related to other EVSs reported in Table 2 (e.g. flashes of bright light, small bright dots). Moreover, we know from our clinical experience that other positive EVS can be flickering (high-frequency micro-movements), such as zigzag lines and round forms. Therefore, we believe that the flickering quality as well as the scintillating quality (high-frequency changes of intensity of light) should be assessed for every EVS (or at least the positive ones) in a prospective study.

In general, the heterogeneity and the limitations in the methodology of the low number of studies that have investigated the features of visual aura, is problematic both for research and clinical practise.

### Visual aura symptoms in a clinical context

MA is a risk factor for several serious cardiovascular conditions, including ischemic and haemorrhagic stroke [6, 7], myocardial infarction [7], atrial fibrillation [8] and perioperative stroke [30]. Moreover, the risk of vascular incidents increases up to 13 fold with the use of combined oral contraceptives [31], which is important considering that the majority of migraine patients are women of reproductive age [32, 33]. Furthermore, the differential diagnosis includes cerebrovascular disorders, epilepsy, and other life-threatening neurological conditions. In clinical practice, MA can be very difficult to distinguish from transient ischemic attacks and stroke. Migraine is the third most common stroke mimic, following seizures and psychiatric disorders, and accounts for 18% of all improper thrombolytic treatment [10]. Vice versa, patients with overlooked strokes in an emergency department setting most often receive an initial misdiagnosis of “migraine” [34].

Therefore, it is of utmost importance to correctly diagnose patients with MA in order to effectively distinguish MA from other, potentially life-threatening, conditions. Indeed, the quality of VASs is one of the most important features and we believe that establishing a consensus in the form of an official list of all MA visual symptoms is essential.

### Future perspectives

After reviewing all data, we have made a list of the 30 EVSs described in clinical studies so far. After that, we created a new list that can be used in future clinical studies (Table 3). We decreased the number of EVSs from 30 to 25 as: i) we put together eight items as we felt they were difficult to distinguish (namely “blind spots” and “black dots”, “micropsia” and “things look closer than they really are”, macropsia and “things look farther away than they really are”, “slanted vision” and “deformed images”) and we deleted “flickering lights”, as “flickering” (as well as “scintillation”) is a feature that can be used to describe other positive EVSs reported in Table 2. Therefore, we propose that some EVSs should be further characterized by determining the presence of “scintillation” and/or “flickering” (Table 2). In addition, we propose that some EVS should be further characterized by their colour and “internal pattern” (Table 2).

**Table 3:** Proposed list of all EVS of migraine aura and their description

Proposed Name	Description
1. Bright light^c^	Single area of bright light
2. Foggy/blurred vision	Foggy or blurred vision
3. Zigzag lines^ac^	Zigzag or jagged lines
4. Scotoma	Single blind area
5. Scotomata	Several blind/black areas
6. Small bright dots^c^	Small bright dots/stars
7. White dots/round forms^abc^	Medium sized white dots/round forms
8. Colored dots/round forms^abc^	Medium sized coloured dots/round forms
9. Lines (colored lines)^abc^	Lines (colored lines)
10. Geometrical shapes^abc^	Geometrical shapes
11. ’Like looking through heat waves, water or oil’	’Like looking through heat waves, water or oil’
12. Visual snow	Dynamic, continuous, tiny dots usually black/gray on white background and gray/white on black background
13. ‘Bean-like’ forms^abc^	‘Bean-like’ forms like a crescent or C-shaped
14. Hemianopsia	Blindness of half of the visual field
15. Deformed images	Deformed images (alteration of lines/angles)
16. Tunnel vision	Blindness in the whole periphery
17. Oscillopsia	Movement of stationary objects
18. Mosaic vision	Seeing mosaic-like
19. Fractured objects	Seeing fractured objects
20. Corona effect^abc^	An extra edge on objects
21. Anopia	Total blindness
22. Micropsia	Objects appear smaller or more distant than they actually are
23. Macropsia	Objects appear larger or closer than they actually are
24. Like a negative film	Seeing like a negative film
25. Complex hallucinations^ac^	Visual perception of something not present (e.g. objects, animals, and persons)

For some EVSs, when reported, patients should be asked about some additional features: ^a^ colour; ^b^ internal pattern (suggested text: “If the inside of the EVS does not have a homogeneous color but is made up an internal pattern (for example zigzag lines or chessboard) please describe it in words”); ^c^ scintillation / flickering (suggested text: “Is/are EVS scintillating (like stars or intermittent lights) and/or flickering (as rapid movements like the wings of a butterfly)?”)

The next step will be to apply this list to clinical studies (1) in patients with MA to better assess the frequency of the different EVSs, thereby establishing which EVSs are the most prevalent and which are clinically meaningful to include in a consensus list; (2) in patients with other visual disturbances (either due to CNS or ocular disorders) to assess the specificity and sensibility of each EVS with respect to MA. This improved description of VASs is important to increase the understanding of other aspects of MA. Pathophysiological studies, e.g. involving neuroimaging and neurophysiology, would benefit from an improved endophenotyping of patients. As an example, a recent functional MRI study suggested that different types of migraine VASs (negative vs. positive) correspond to different types of cerebral dysfunction [35]. Different VAS phenotypes may therefore prove to differ in terms of prognosis, risk of cardiovascular disease, and response to treatment.

## Conclusion

We created a comprehensive list of VASs reported by migraine patients based on all currently available data from clinical studies. The most frequently reported symptoms were flashes of bright light, “foggy” vision, zigzag lines, and scotoma. We observed a lack of prospective studies and a relatively high degree of discrepancy between studies, likely mostly due to differences in the terminology used to describe VASs. We emphasize the importance of an improved classification of migraine VASs and propose that an official list of visual symptoms should be developed for this purpose, preferably within the context of the International Classification of Headache Disorders of the International Headache Society.

## Abbreviations

EVS: Elementary visual symptom
ICHD: International Classification of Headache Disorders
MA: Migraine aura
VAS: Visual aura symptom
